# Sensory evaluation of edoxaban orally disintegrating tablets: an open-label interventional study (secondary publication)

**DOI:** 10.1186/s12959-019-0192-x

**Published:** 2019-02-15

**Authors:** Takeshi Yamashita, Joji Hagii, Yoshiyuki Morishima, Takaaki Akasaka, Takuyuki Matsumoto, Tetsuya Kimura

**Affiliations:** 1 The Cardiovascular Institute, 3-2-19, Nishiazabu, Minato-ku, Tokyo 106-0031 Japan; 2 Hirosaki Stroke and Rehabilitation Center, 1-2-1, Ougi-machi, Hirosaki, Aomori 036-8104 Japan; 3 Daiichi Sankyo Co., Ltd., 3-5-1, Nihonbashi Honcho, Chuo-ku, Tokyo 103-8426 Japan

**Keywords:** Edoxaban, Nonvalvular atrial fibrillation, Orally disintegrating (OD) tablet, Sensory evaluation

## Abstract

**Background:**

This study involved a sensory evaluation of edoxaban orally disintegrating (OD) tablets in patients with nonvalvular atrial fibrillation who had been receiving the existing edoxaban film-coated tablets before the study.

**Methods:**

Edoxaban OD tablets 30 or 60 mg were prescribed for patients who had been receiving the existing 30- or 60-mg edoxaban film-coated tablets before the study. Each dose group was randomized into groups taking the tablets with or without water. After ingestion of the edoxaban OD tablet, each patient was asked to complete a sensory evaluation questionnaire (12 items).

**Results:**

In the evaluation of satisfaction with edoxaban OD tablets, 52.8% of the patients perceived “no difference” from the existing edoxaban film-coated tablets and 34.9% indicated that they were more satisfied with the OD tablets, thus demonstrating a relatively high degree of satisfaction. When asked about convenience and reliability in using edoxaban OD tablets, about half of the patients perceived “no difference” from the existing edoxaban film-coated tablets and the remaining half indicated preference for the OD tablets. Responses about taste, flavor, ease of ingestion, and motivation to continue taking edoxaban indicated the overall acceptance of the OD tablets. Recognition of edoxaban OD tablets was rated as “easy” by about half of the patients and “difficult” by the remaining half. Among all patients, 49.5% preferred a change to edoxaban OD tablets. The degree of satisfaction with taste, flavor, and ease of ingestion, as well as overall satisfaction, tended to be greater when the OD tablets were taken with rather than without water, and the percentage of patients who preferred a change was higher in the group taking the OD tablets with water.

**Conclusions:**

This study indicated that the degree of satisfaction with taste, flavor, ease of ingestion, and convenience, as well as overall satisfaction, in addition to motivation to continue drug intake and sense of confidence were greater for OD tablets than for the existing edoxaban film-coated tablets. Edoxaban OD tablet is a promising formulation for inducing greater patient adherence to medication and therefore ensures better treatment response.

**Trial registration:**

UMIN-CTR UMIN000028788, registered 23-Aug-2017.

**Electronic supplementary material:**

The online version of this article (10.1186/s12959-019-0192-x) contains supplementary material, which is available to authorized users.

## Background

Some patients with diseases requiring anticoagulant therapy such as atrial fibrillation have impaired swallowing function due to reasons including advanced age and history of stroke [1]. Decreased medication adherence in these patients could be attributable to difficulty of taking standard tablets. Improvement of medication adherence is essential for a safe and effective anticoagulant therapy, as anticoagulants require long-term use. Use of orally disintegrating (OD) tablets is one way of improving medication adherence. OD tablets are easy to be taken by patients with restricted water ingestion, the elderly, and those with difficulty swallowing because they readily disintegrate in the oral cavity and can be taken anywhere without water. Thus, the use of OD tablets is expected to improve medication adherence, which results in improved therapeutic performance. Patients who changed from standard tablets to OD tablets have been reported to show improvement of medication adherence and therapeutic efficacy [2, 3].

Edoxaban (product name in Japan: Lixiana®) is a direct oral anticoagulant (DOAC) with indications for prevention of stroke and systemic embolism in patients with nonvalvular atrial fibrillation and treatment and prevention of recurrent venous thromboembolism, among others. Since 2017, edoxaban OD tablet, which is the first OD tablet formulation of DOACs, has been marketed in Japan.

This questionnaire survey was conducted for sensory evaluation of edoxaban OD tablets (a single dose of 30 or 60 mg) to assess the impression upon ingestion, sense of convenience, ease of recognition as edoxaban OD tablets, and desire to switch to edoxaban OD tablets in patients with nonvalvular atrial fibrillation who had been receiving existing edoxaban film-coated tablets (30 or 60 mg) before the study.

## Methods

### Patients

This study included 108 patients aged ≥20 years who had nonvalvular atrial fibrillation and visited the Cardiovascular Institute Hospital or Hirosaki Stroke and Rehabilitation Center between August 2017 and January 2018. All patients had been receiving 30- or 60-mg edoxaban film-coated tablets for ≥2 weeks, and provided written consent to participate in this study.

The following patients were excluded: those receiving dual antiplatelet therapy; those with a cardiovascular event (stroke, myocardial infarction, cardiac intervention other than myocardial infarction, or heart failure requiring hospitalization) or those hospitalized for bleeding within 2 weeks before enrollment; or those who were pregnant, lactating, or possibly pregnant.

### Study design

This was an open-label interventional study, with patients given a single dose of one edoxaban OD tablet (30 or 60 mg).

On the day of the visit, each patient underwent blood sampling for measuring prothrombin time to confirm that no edoxaban tablet had been taken that day. A corresponding 30- or 60-mg edoxaban OD tablet was prescribed for each patient who had been receiving 30- or 60-mg edoxaban film-coated tablets before the start of the study. Each of these 2 groups was subdivided randomly into 2 groups (taking an OD tablet with or without water) in an allocation ratio of 1:1 (4 groups in total). A dynamic allocation was performed using study site, age at enrollment (≥75 or < 75 years), and sex as adjustment factors to avoid their influence on outcome. Simultaneous ingestion of edoxaban OD tablet with any other drugs was prohibited. The target total number of participants was 100 patients in 4 groups (set at 20 or 30 patients/group), in accordance with previous clinical studies on sensory evaluation of OD tablets [4–6].

After ingestion of the 30- or 60-mg edoxaban OD tablet, each patient was asked to answer all 12 questions in the sensory evaluation questionnaire (Table 1), and the answers to each question were analyzed. An open-ended answer was permitted for the “Reason for the selected answer.”

**Table 1:** Sensory evaluation questionnaire

	Question										
Please answer the questions about the drugs you are routinely taking.											
Question 1	Have you ever found difficulty taking any of the tablets you are routinely taking?										
	1. Yes 2. No										
Question 2	Have you ever been unable to identify the drug taken out of the sheet, causing you to stop taking the drug or any other difficulty?										
	1. Yes 2. No										
Question 3	How many types of drugs are you routinely taking every morning, including the edoxaban film-coated tablet?										
	[types]										
Next, please answer the questions about the edoxaban orally disintegrating (OD) tablet you have just taken.											
Question 4	What is the extent of your satisfaction with the edoxaban OD tablet you have just taken, if your satisfaction with the current medication (edoxaban film-coated tablet) rates 5?										
	Quite unsatisfactory	No difference									Quite satisfactory
	0	1	2	3	4	5	6	7	8	9	10
Question 5	The product name and dose level are printed on this tablet. Was it easy for you to identify that the drug was edoxaban OD tablet 30 mg or 60 mg?										
	1. Quite easy to identify										
	2. Easier to identify than the current edoxaban film-coated tablet										
	3. More difficult to identify than the current edoxaban film-coated tablet										
	4. Quite difficult to identify										
Question 6	What was your impression about the size of this tablet when you placed it in your mouth?										
	1. Very large										
	2. Slightly large										
	3. Slightly small										
	4. Very small										
Question 7	What was your impression of the taste and flavor of this drug when it disintegrated in your mouth?										
	1. Very good										
	2. Relatively good										
	3. Relatively bad (i: Tolerable, ii: Not tolerable)										
	4. Very bad (i: Tolerable, ii: Not tolerable)										
	→Please give the reason for selecting 1 through 4, if possible: [Reason:]										
Question 8	Have you found this drug easier to take than the current medication (edoxaban film-coated tablet)?										
	1. Quite easy to take										
	2. Slightly easier to take										
	3. No difference										
	4. Slightly more difficult to take										
	5. Quite difficult to take										
	→Please give the reason for selecting 1 through 5, if possible: [Reason:]										
This drug can be taken both with or without water, and simultaneously with other drugs. The product name and dose level are printed on this tablet.											
Question 9	Do you think that this drug is more convenient to take than the current medication (edoxaban film-coated tablet)?										
	1. Much more convenient										
	2. Slightly more convenient										
	3. No difference										
	4. Slightly less convenient										
	5. Much less convenient										
	→Please give the reason for selecting 1 through 5, if possible: [Reason:]										
Question 10	Do you think this drug is easier to continue than the current medication (edoxaban film-coated tablet)?										
	1. Much easier to continue										
	2. Slightly easier to continue										
	3. No difference										
	4. Slightly less easy to continue										
	5. Much less easy to continue										
	→Please give the reason for selecting 1 through 5, if possible: [Reason:]										
Question 11	Do you think that the printed product name and dose level on the tablet make this drug more reliable than the current medication (edoxaban film-coated tablet)?										
	1. Much more reliable										
	2. Slightly more reliable										
	3. No difference										
	4. Slightly less reliable										
	5. Much less reliable										
Question 12	This drug will be sold at the same price as the current medication (edoxaban film-coated tablet). Will you want to change to this drug after marketed?										
	1. Desire to change										
	2. No desire to change										
	→Please give the reason for selecting 1 or 2, if possible: [Reason:]										

Safety was assessed by recording any adverse events (AEs) from immediately after ingestion of edoxaban OD tablets to the following day.

The protocol was registered with the open database UMIN Clinical Trials Registry (Trial ID: UMIN000028788) prior to the start of the study, and the summarized results were also registered after completion of the study.

### Statistical analyses

For patient background characteristics and answers to the sensory evaluation questionnaire (12 items), summary statistics for continuous data and numbers and percentages for categorical data were calculated. For safety evaluation, the number of patients with AEs or adverse drug reactions (ADRs) was counted.

These analyses were conducted for the subgroups (age < 75 or ≥ 75 years; presence or absence of difficulty taking the current medication). These statistical analyses were performed using SAS Ver. 9.4.

## Results

In total, 108 patients were enrolled and allocated for this study. The 66 patients who were receiving 30-mg edoxaban film-coated tablets before the start of the study were allocated to group 1 (30-mg edoxaban OD tablet without water; *n* = 33) and group 3 (30-mg edoxaban OD tablet with water; *n* = 33). The 42 patients who had been receiving 60-mg edoxaban film-coated tablets before the start of the study were allocated to group 2 (60-mg edoxaban OD tablet without water; *n* = 21) and group 4 (60-mg edoxaban OD tablet with water; *n* = 21).

Nine patients (group 1, *n* = 4; group 3, *n* = 3; and group 4, *n* = 2) discontinued the study because of violation of the inclusion/exclusion criteria (*n* = 8) and cancellation of the study drug administration at the discretion of the physician (*n* = 1). The efficacy analysis set included 106 of 108 patients, excluding 1 patient with a serious violation of the study protocol and 1 patient in whom the administration was cancelled at the discretion of the physician. The safety analysis set included 107 patients, excluding 1 patient in whom the administration was cancelled at the discretion of the physician.

### Background characteristics

Table 2 shows the background characteristics of the efficacy analysis set (*n* = 106). The patients’ mean (± standard deviation) age was 73.0 ± 8.6 years. Of the patients, 49.1% (52/106) were aged ≥75 years and 60.4% (64/106) were men. For the 79 patients who had data on height, the mean height was 161.6 ± 10.0 cm. The mean body weight was 61.1 ± 13.5 kg.

**Table 2:** Background characteristics (efficacy analysis set)

		n	Mean ± SD or %
Age, y, mean ± SD	Total	106	73.0 ± 8.6
	Group 1 (30 mg without water)^a^	32	77.0 ± 6.2
	Group 3 (30 mg with water)^b^	32	76.7 ± 6.3
	Group 2 (60 mg without water)^c^	21	67.7 ± 8.9
	Group 4 (60 mg with water)^d^	21	66.9 ± 8.3
Age (≥75 years old), %	Total	52	49.1
	Group 1 (30 mg without water)^a^	21	65.6
	Group 3 (30 mg with water)^b^	20	62.5
	Group 2 (60 mg without water)^c^	6	28.6
	Group 4 (60 mg with water)^d^	5	23.8
Male, %	Total	64	60.4
	Group 1 (30 mg without water)^a^	11	34.4
	Group 3 (30 mg with water)^b^	12	37.5
	Group 2 (60 mg without water)^c^	21	100.0
	Group 4 (60 mg with water)^d^	20	95.2
Height, cm, mean ± SD	Total	79	161.64 ± 9.97
	Group 1 (30 mg without water)^a^	24	156.79 ± 8.43
	Group 3 (30 mg with water)^b^	24	156.25 ± 7.19
	Group 2 (60 mg without water)^c^	17	170.69 ± 7.93
	Group 4 (60 mg with water)^d^	14	168.19 ± 7.22
Body weight, kg, mean ± SD	Total	106	61.12 ± 13.49
	Group 1 (30 mg without water)^a^	32	52.69 ± 7.38
	Group 3 (30 mg with water)^b^	32	52.80 ± 6.71
	Group 2 (60 mg without water)^c^	21	71.85 ± 11.13
	Group 4 (60 mg with water)^d^	21	75.91 ± 10.09
Prior cerebral infarction/transient ischemic attack, %	Total	46	43.4
	Group 1 (30 mg without water)^a^	17	53.1
	Group 3 (30 mg with water)^b^	14	43.8
	Group 2 (60 mg without water)^c^	8	38.1
	Group 4 (60 mg with water)^d^	7	33.3

^a^Edoxaban OD tablet 30 mg without water

^b^edoxaban OD tablet 30 mg with water

^c^edoxaban OD tablet 60 mg without water

^d^edoxaban OD tablet 60 mg with water

*OD* orally disintegrating, *SD* standard deviation

### Sensory evaluation questionnaire

**Fig. 1:**
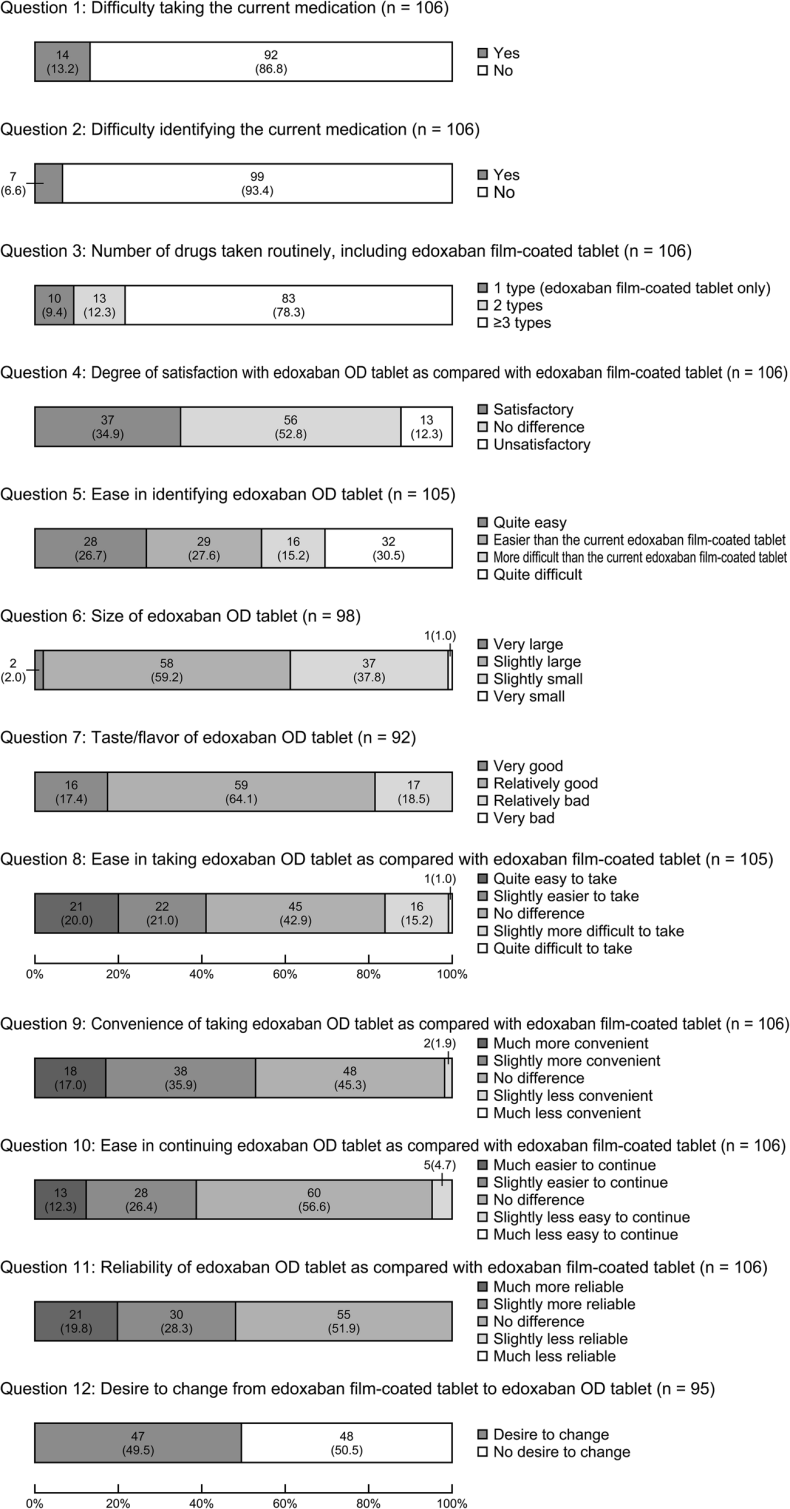
Results of the sensory evaluation questionnaire survey on edoxaban OD tablets. Values in the bar graph represents the number of patients (%). *OD* orally disintegrating

For the efficacy analysis set (*n* = 106), the responses to the sensory evaluation questionnaire for the total and subgroups are shown in Fig. 1 and Table 3, respectively. Questions 1–3 pertain to the current medication, and questions 4–12 pertain to edoxaban OD tablets.

**Table 3:** Results of the sensory evaluation questionnaire about edoxaban OD tablets shown by the group

		Group 1 (30 mg without water)^c^ *N* = 32	Group 3 (30 mg with water)^d^ *N* = 32	Group 2 (60 mg without water)^e^ *N* = 21	Group 4 (60 mg with water)^f^ *N* = 21
		n (%)	n (%)	n (%)	n (%)
Question 1: Difficulty in ingestion (current)	Yes	6 (18.8)	5 (15.6)	2 (9.5)	1 (4.8)
	No	26 (81.3)	27 (84.4)	19 (90.5)	20 (95.2)
Question 2: Difficulty in identifying (current)	Yes	2 (6.3)	3 (9.4)	0 (0.0)	2 (9.5)
	No	30 (93.8)	29 (90.6)	21 (100.0)	19 (90.5)
Question 3: Number of drugs taken simultaneously	1 type^a^	3 (9.4)	3 (9.4)	1 (4.8)	3 (14.3)
	2 types	6 (18.8)	2 (6.3)	4 (19.1)	1 (4.8)
	≥3 types	23 (71.9)	27 (84.4)	16 (76.2)	17 (81.0)
Question 4: Degree of satisfaction	Unsatisfactory	5 (15.6)	1 (3.1)	7 (33.3)	0 (0.0)
	No difference	15 (46.9)	21 (65.6)	6 (28.6)	14 (66.7)
	Satisfactory	12 (37.5)	10 (31.3)	8 (38.1)	7 (33.3)
Question 5: Ease in identifying	Quite easy	7 (21.9)	7 (21.9)	6 (30.0)	8 (38.1)
	Easier^b^	8 (25.0)	11 (34.4)	7 (35.0)	3 (14.3)
	More difficult^b^	5 (15.6)	3 (9.4)	4 (20.0)	4 (19.1)
	Quite difficult	12 (37.5)	11 (34.4)	3 (15.0)	6 (28.6)
Question 6: Size	Very large	1 (3.6)	0 (0.0)	0 (0.0)	1 (5.0)
	Slightly large	10 (35.7)	19 (61.3)	16 (84.2)	13 (65.0)
	Slightly small	16 (57.1)	12 (38.7)	3 (15.8)	6 (30.0)
	Very small	1 (3.6)	0 (0.0)	0 (0.0)	0 (0.0)
Question 7: Taste at the time of disintegration	Very good	5 (16.7)	5 (20.0)	1 (5.3)	5 (27.8)
	Relatively good	16 (53.3)	19 (76.0)	12 (63.2)	12 (66.7)
	Relatively bad	9 (30.0)	1 (4.0)	6 (31.6)	1 (5.6)
	Very bad	0 (0.0)	0 (0.0)	0 (0.0)	0 (0.0)
Question 8: Ease in ingestion (comparison)	Quite easy to take	8 (25.0)	8 (25.8)	2 (9.5)	3 (14.3)
	Slightly easier to take	6 (18.8)	8 (25.8)	3 (14.3)	5 (23.8)
	No difference	9 (28.1)	15 (48.4)	9 (42.9)	12 (57.1)
	Slightly more difficult to take	8 (25.0)	0 (0.0)	7 (33.3)	1 (4.8)
	Quite difficult to take	1 (3.1)	0 (0.0)	0 (0.0)	0 (0.0)
Question 9: Convenience	Much more convenient	7 (21.9)	3 (9.4)	2 (9.5)	6 (28.6)
	Slightly more convenient	9 (28.1)	15 (46.9)	6 (28.6)	8 (38.1)
	No difference	14 (43.8)	14 (43.8)	13 (61.9)	7 (33.3)
	Slightly less convenient	2 (6.3)	0 (0.0)	0 (0.0)	0 (0.0)
	Much less convenient	0 (0.0)	0 (0.0)	0 (0.0)	0 (0.0)
Question 10: Ease of continuation	Much easier to continue	6 (18.8)	2 (6.3)	1 (4.8)	4 (19.1)
	Slightly easier to continue	6 (18.8)	13 (40.6)	6 (28.6)	3 (14.3)
	No difference	17 (53.1)	17 (53.1)	12 (57.1)	14 (66.7)
	Slightly less easy to continue	3 (9.4)	0 (0.0)	2 (9.5)	0 (0.0)
	Much less easy to continue	0 (0.0)	0 (0.0)	0 (0.0)	0 (0.0)
Question 11: Reliability	Much more reliable	2 (6.3)	7 (21.9)	6 (28.6)	6 (28.6)
	Slightly more reliable	11 (34.4)	9 (28.1)	5 (23.8)	5 (23.8)
	No difference	19 (59.4)	16 (50.0)	10 (47.6)	10 (47.6)
	Slightly less reliable	0 (0.0)	0 (0.0)	0 (0.0)	0 (0.0)
	Much less reliable	0 (0.0)	0 (0.0)	0 (0.0)	0 (0.0)
Question 12: Desire to change	Desire to change	12 (44.4)	19 (63.3)	6 (30.0)	10 (55.6)
	No desire to change	15 (55.6)	11 (36.7)	14 (70.0)	8 (44.4)

^a^Edoxaban film-coated tablet only

^b^compared with the current medication

^c^edoxaban OD tablet 30 mg without water

^d^edoxaban OD tablet 30 mg with water

^e^edoxaban OD tablet 60 mg without water

^f^edoxaban OD tablet 60 mg with water

*OD* orally disintegrating

#### Current medication

For question 1 (Have you ever found difficulty taking any of the tablets you are routinely taking?), 13.2% (14/106) of the respondents answered “Yes.”

For question 2 (Have you ever been unable to identify the drug taken out of the sheet, causing you to stop taking the drug or any other difficulty?), 6.6% (7/106) selected “Yes.”

For question 3 (How many types of drugs are you routinely taking every morning, including the edoxaban film-coated tablet?), “three types or more” was selected most frequently (78.3%, 83/106), followed by “2 types” (12.3%, 13/106) and “1 type (edoxaban film-coated tablet alone)” (9.4%, 10/106).

#### Edoxaban OD tablets

**Fig. 2:**
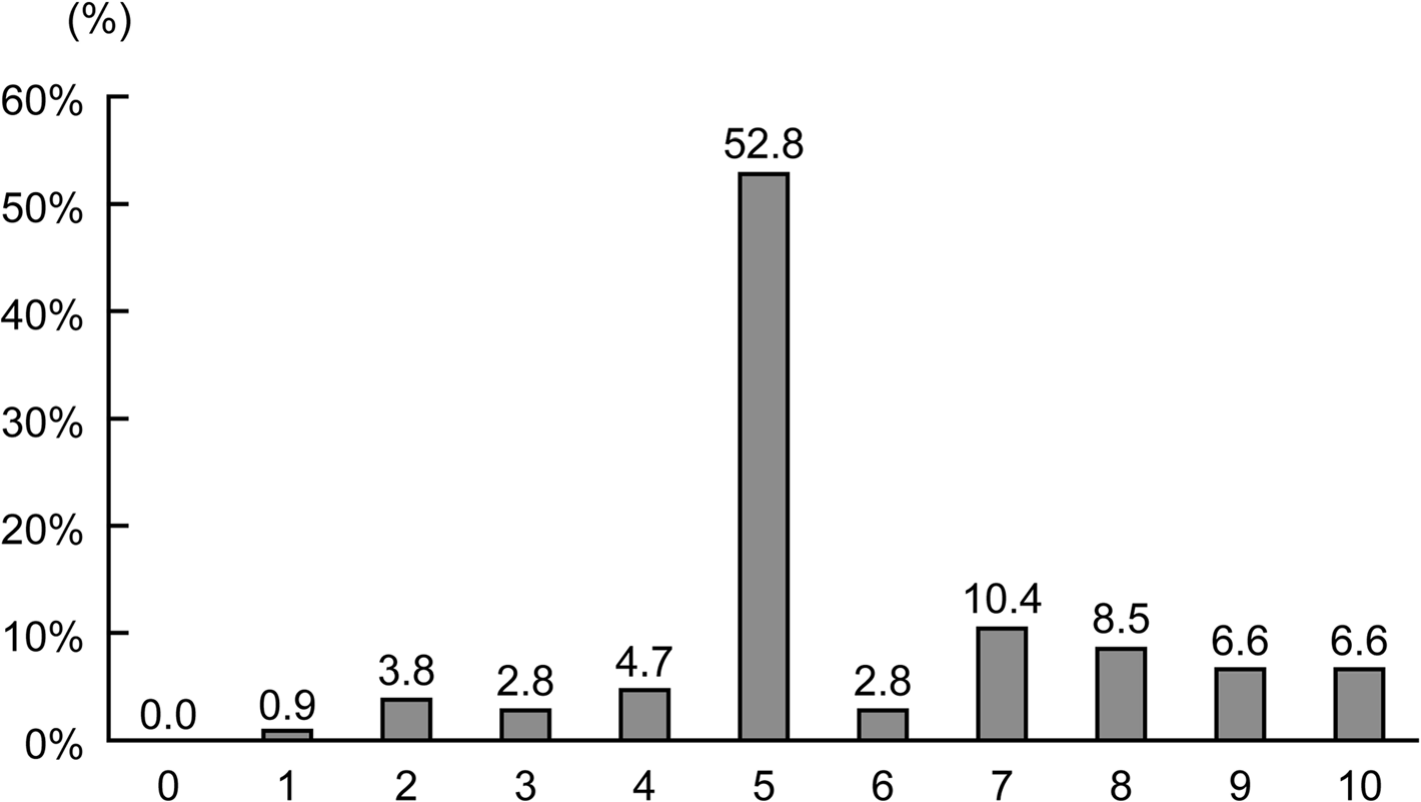
The histogram of the degree of satisfaction with edoxaban OD tablets (question 4). The graph represents the frequency of patients in each answer (11-grade scale) to the question 4 of the sensory evaluation questionnaire. 0: Quite unsatisfactory, 5: no difference, and 10: quite satisfactory. *OD* orally disintegrating

For question 4 (What is the extent of your satisfaction with the edoxaban OD tablet you have just taken, if your satisfaction with the current medication [edoxaban film-coated tablet] rates 5?), the answers on the 11-grade scale are shown in Fig. 2. “No difference (rating: 5)” was selected most frequently (52.8%, 56/106), followed by “satisfactory (rating: 6–10)” (34.9%, 37/106) and “unsatisfactory (rating: 0–4)” (12.3%, 13/106) (Fig. 1).

For question 5 (The product name and dose level are printed on this tablet. Was it easy for you to identify that the drug was edoxaban OD tablet 30 mg or 60 mg?), “quite difficult to identify” was selected most frequently (30.5%, 32/105), followed by “easier to identify than the current edoxaban film-coated tablet” (27.6%, 29/105), “quite easy to identify” (26.7%, 28/105), and “more difficult to identify than the current edoxaban film-coated tablet” (15.2%, 16/105).

For question 6 (What was your impression about the size of this tablet when you placed it in your mouth?), “slightly large” was selected most frequently (59.2%, 58/98), followed by “slightly small” (37.8%, 37/98), “very large” (2.0%, 2/98), and “very small” (1.0%, 1/98).

For question 7 (What was your impression of the taste and flavor of this drug when it disintegrated in your mouth?), “relatively good” was selected most frequently (64.1%, 59/92), followed by “relatively bad” (18.5%, 17/92) and “very good” (17.4%, 16/92). “Very bad” was not selected by any of the patients. Of the patients who selected “relatively bad,” 15 stated that it was “tolerable” and 1 stated that it was “not tolerable.” Of the patients who selected “relatively bad,” 15 took the tablet without water. The major reasons for responding “good” were “no concerns,” “easy to swallow,” and “slightly sweet and smoothly dissolving,” and those for selecting “relatively bad” were “bitter,” “sour,” “not dissolving smoothly,” and “slightly starchy.”

For question 8 (Have you found this drug easier to take than the current medication [edoxaban film-coated tablet]?), “no difference” was selected most frequently (42.9%, 45/105), followed by “slightly easier to take” (21.0%, 22/105), “quite easy to take” (20.0%, 21/105), “slightly more difficult to take” (15.2%, 16/105), and “quite difficult to take” (1.0%, 1/105). Of the patients who selected “slightly more difficult to take” or “quite difficult to take,” 16 took the tablet without water. The major reasons for selecting “easy to take” were “sweet,” “tasty,” and “smoothly dissolving,” and those for selecting “difficult to take” were “easier to take with water,” “bitter,” and “not smoothly dissolving.”

For question 9 (Do you think that this drug is more convenient to take than the current medication [edoxaban film-coated tablet]?), “no difference” was selected most frequently (45.3%, 48/106), followed by “slightly more convenient” (35.9%, 38/106), “much more convenient” (17.0%, 18/106), and “slightly less convenient” (1.9%, 2/106). “Much less convenient” was not selected by any of the patients. The major reasons for selecting “more convenient” were “can be taken without water” and “does not require effort to swallow,” and those for selecting “no difference” was “I take it with water, simultaneously with other drugs.”

For question 10 (Do you think this drug is easier to continue than the current medication [edoxaban film-coated tablet]?), “no difference” was selected most frequently (56.6%, 60/106), followed by “slightly easier to continue” (26.4%, 28/106), “much easier to continue” (12.3%, 13/106), and “slightly less easy to continue” (4.7%, 5/106). “Much less easy to continue” was not selected by any of the patients. The major reasons for selecting “easier to continue” were “can be taken without water” and “the smoothly dissolving nature may be advantageous,” and those for selecting “no difference” were “both existing film-coated tablet and OD tablet are easy to take” and “finds no difference,” and those for selecting “slightly less easy to continue” were “bitter” and “it is better when taken with water.”

For question 11 (Do you think that the printed product name and dose level on the tablet make this drug more reliable than the current medication [edoxaban film-coated tablet]?), “no difference” was selected most frequently (51.9%, 55/106), followed by “slightly more reliable” (28.3%, 30/106) and “much more reliable” (19.8%, 21/106). “Slightly less reliable” and “much less reliable” were not selected by any of the patients.

For question 12 (This drug will be sold at the same price as the current medication [edoxaban film-coated tablet]. Will you want to change to this drug after marketed?), “No desire to change” was selected by 50.5% (48/95) and “desire to change” was selected by 49.5% (47/95). The major reasons for “no desire to change” were “water is needed when taking it together with other drugs,” “I have some other drugs to take simultaneously,” “it does not taste good,” and others, and those for selecting “desire to change” were “easier to take,” “less likely to incorrectly identify,” and others.

#### Comparison of sensory evaluation between ingestion with and without water

**Fig. 3:**
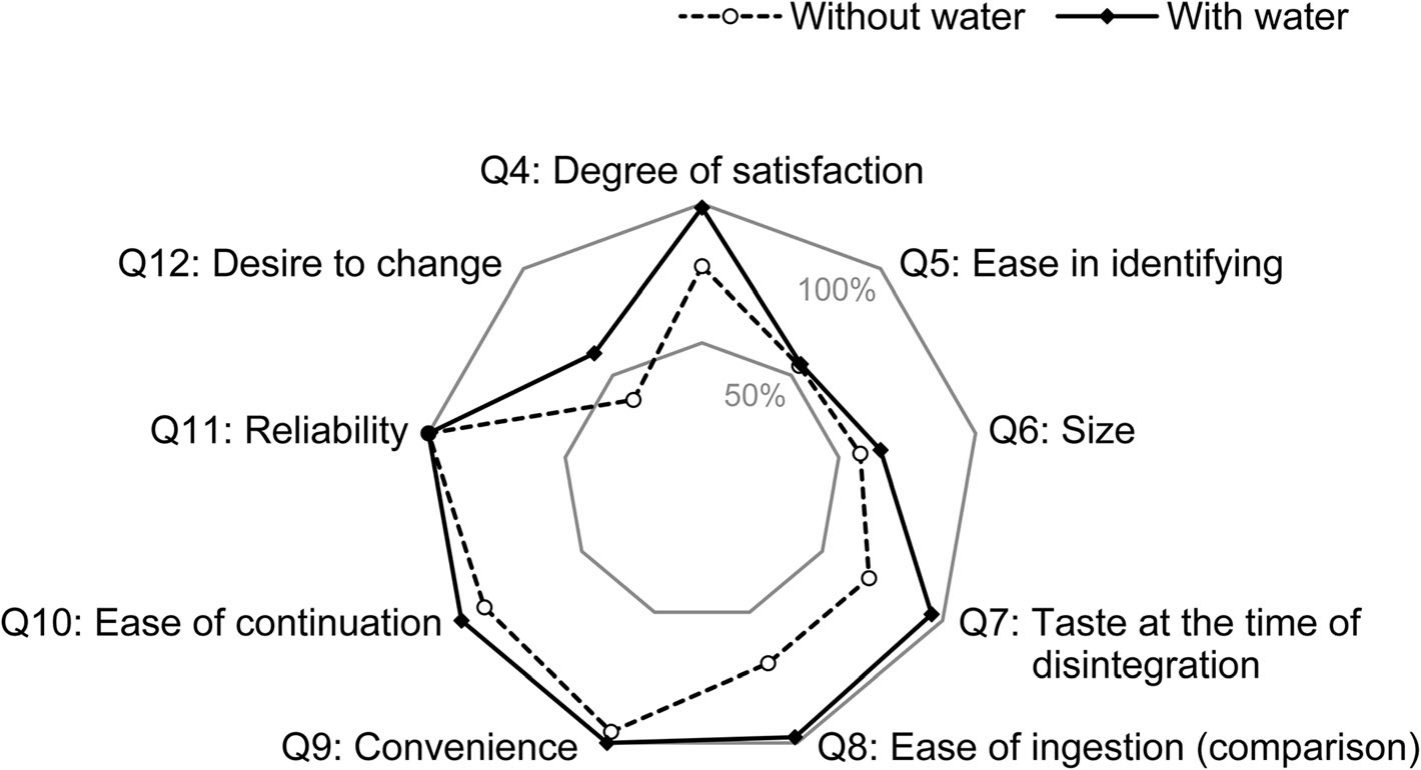
Comparison of sensory evaluation between groups that ingested edoxaban OD tablets with or without water. The graph shows the percentages of patients who answered “5” (no difference) through “10” (quite satisfactory) in question 4; “1” (very good) or “2” (relatively good) in questions 5 and 7; “1” (very large) or “2” (slightly large) in question 6; “1” (very good) through “3” (no difference) in questions 8–11; and “1” (desire to change) in question 12. *OD* orally disintegrating, *Q* question

The comparison of sensory evaluation between ingestion without water (groups 1 and 2) and with water (groups 3 and 4) is shown in Fig. 3 and Table S1 (Additional file 1). Figure 3 shows the percentages of patients who answered “5” (no difference) through “10” (quite satisfactory) in question 4; “1” (very good) or “2” (relatively good) in questions 5 and 7; “1” (very large) or “2” (slightly large) in question 6; “1” (very good) through “3” (no difference) in questions 8–11; and “1” (desire to change) in question 12. The major items found to differ between ingestion with and without water were the degree of satisfaction (question 4), taste and flavor at the time of edoxaban OD tablet disintegration (question 7), ease of ingestion (question 8), and desire a change to edoxaban OD tablets from the current medication (question 12).

“Unsatisfactory” for question 4 (degree of satisfaction) was selected by 1.9% (1/53) and 22.6% (12/53) of the patients who ingested edoxaban OD tablets with and without water, respectively. “Relatively bad” for question 7 (taste and flavor) was selected by 4.7% (2/43) and 30.6% (15/49) of the patients who ingested edoxaban OD tablets with and without water, respectively. “Slightly more difficult to take” or “quite difficult to take” for question 8 (ease of ingestion compared with edoxaban film-coated tablets) was selected by 1.9% (1/52) and 30.2% (16/53) of the patients who ingested the drug with and without water, respectively. “Desire to change” to the OD tablet for question 12 was selected by 60.4% (29/48) and 38.3% (18/47) of the patients who ingested the drug with and without water, respectively.

### Subgroup analysis

Table 4 shows the results of subgroup analysis of sensory evaluation of edoxaban OD tablets by age (< 75 and ≥ 75 years) and response to question 1 (difficulty taking the current medication).

**Table 4:** Results of the sensory evaluation questionnaire about edoxaban OD tablets shown by subgroup

		Age (< 75 years) *N* = 54	Age (≥75 years) *N* = 52	Current difficulty in ingestion (Question 1)	
				Yes *N* = 14	No *N* = 92
		n (%)	n (%)	n (%)	n (%)
Question 1: Difficulty in ingestion (current)	Yes	6 (11.1)	8 (15.4)	14 (100.0)	0 (0.0)
	No	48 (88.9)	44 (84.6)	0 (0.0)	92 (100.0)
Question 2: Difficulty in identifying (current)	Yes	2 (3.7)	5 (9.6)	2 (14.3)	5 (5.4)
	No	52 (96.3)	47 (90.4)	12 (85.7)	87 (94.6)
Question 3: Number of drugs taken simultaneously	1 type^a^	2 (3.7)	8 (15.4)	1 (7.1)	9 (9.8)
	2 types	7 (13.0)	6 (11.5)	2 (14.3)	11 (12.0)
	≥3 types	45 (83.3)	38 (73.1)	11 (78.6)	72 (78.3)
Question 4: Degree of satisfaction	Unsatisfactory	7 (13.0)	6 (11.5)	1 (7.1)	12 (13.0)
	No difference	27 (50.0)	29 (55.8)	6 (42.9)	50 (54.4)
	Satisfactory	20 (37.0)	17 (32.7)	7 (50.0)	30 (32.6)
Question 5: Ease in identifying	Quite easy	17 (31.5)	11 (21.6)	5 (35.7)	23 (25.3)
	Easier^b^	13 (24.1)	16 (31.4)	5 (35.7)	24 (26.4)
	More difficult^b^	11 (20.4)	5 (9.8)	1 (7.1)	15 (16.5)
	Quite difficult	13 (24.1)	19 (37.3)	3 (21.4)	29 (31.9)
Question 6: Size	Very large	2 (3.8)	0 (0.0)	0 (0.0)	2 (2.4)
	Slightly large	34 (64.2)	24 (53.3)	7 (53.9)	51 (60.0)
	Slightly small	17 (32.1)	20 (44.4)	6 (46.2)	31 (36.5)
	Very small	0 (0.0)	1 (2.2)	0 (0.0)	1 (1.2)
Question 7: Taste at the time of disintegration	Very good	9 (18.4)	7 (16.3)	2 (14.3)	14 (18.0)
	Relatively good	31 (63.3)	28 (65.1)	11 (78.6)	48 (61.5)
	Relatively bad	9 (18.4)	8 (18.6)	1 (7.1)	16 (20.5)
	Very bad	0 (0.0)	0 (0.0)	0 (0.0)	0 (0.0)
Question 8: Ease of ingestion (comparison)	Quite easy to take	10 (18.9)	11 (21.2)	6 (42.9)	15 (16.5)
	Slightly easier to take	14 (26.4)	8 (15.4)	3 (21.4)	19 (20.9)
	No difference	22 (41.5)	23 (44.2)	3 (21.4)	42 (46.2)
	Slightly more difficult to take	6 (11.3)	10 (19.2)	2 (14.3)	14 (15.4)
	Quite difficult to take	1 (1.9)	0 (0.0)	0 (0.0)	1 (1.1)
Question 9: Convenience	Much more convenient	8 (14.8)	10 (19.2)	4 (28.6)	14 (15.2)
	Slightly more convenient	22 (40.7)	16 (30.8)	6 (42.9)	32 (34.8)
	No difference	23 (42.6)	25 (48.1)	4 (28.6)	44 (47.8)
	Slightly less convenient	1 (1.9)	1 (1.9)	0 (0.0)	2 (2.2)
	Much less convenient	0 (0.0)	0 (0.0)	0 (0.0)	0 (0.0)
Question 10: Ease of continuation	Much easier to continue	6 (11.1)	7 (13.5)	5 (35.7)	8 (8.7)
	Slightly easier to continue	16 (29.6)	12 (23.1)	2 (14.3)	26 (28.3)
	No difference	29 (53.7)	31 (59.6)	7 (50.0)	53 (57.6)
	Slightly less easy to continue	3 (5.6)	2 (3.9)	0 (0.0)	5 (5.4)
	Much less easy to continue	0 (0.0)	0 (0.0)	0 (0.0)	0 (0.0)
Question 11: Reliability	Much more reliable	16 (29.6)	5 (9.6)	5 (35.7)	16 (17.4)
	Slightly more reliable	16 (29.6)	14 (26.9)	2 (14.3)	28 (30.4)
	No difference	22 (40.7)	33 (63.5)	7 (50.0)	48 (52.2)
	Slightly less reliable	0 (0.0)	0 (0.0)	0 (0.0)	0 (0.0)
	Much less reliable	0 (0.0)	0 (0.0)	0 (0.0)	0 (0.0)
Question 12: Desire to change	Desire to change	28 (57.1)	19 (41.3)	10 (76.9)	37 (45.1)
	No desire to change	21 (42.9)	27 (58.7)	3 (23.1)	45 (54.9)

^a^Edoxaban film-coated tablet only

^b^compared with the current medication

*OD* orally disintegrating

#### Age < 75 and ≥ 75 years

The major items found to differ between the < 75 and ≥ 75 year age groups were reliability (question 11) and the desire to change to the OD tablets (question 12). “Much more reliable” was selected by 29.6% (16/54) of the patients aged < 75 years and by 9.6% (5/52) of those aged ≥75 years. “Desire to change” to edoxaban the OD tablets for question 12 was selected by 57.1% (28/49) of the patients aged < 75 years and by 41.3% (19/46) of those aged ≥75 years.

#### Presence/absence of difficulty taking the current medication

The major items found to differ between the patients who perceived difficulty taking the current medication and those who did not perceive difficulty were “ease of ingestion” (question 8), “ease in continuing drug ingestion” (question 10), and “desire change to the OD tablet” (question 12). Regarding the ease of taking edoxaban OD tablets (question 8), “quite easy to take” was selected by 42.9% (6/14) of those who perceived difficulty taking the current medication and 16.5% (15/91) of those who did not. Regarding ease of continuing drug ingestion (question 10), “much easier to continue” was selected by 35.7% (5/14) of those who perceived difficulty taking the current medication and 8.7% (8/92) of those who did not. “Desire to change” to edoxaban OD tablets in question 12 was selected by 76.9% (10/13) of those who perceived difficulty taking the current medication and 45.1% (37/82) of those who did not.

### AEs and ADRs

During the study period, 4 AEs occurred in 4 patients, including palpitation, diarrhea, and chest discomfort (1 event each) in the 30-mg edoxaban OD tablet group and excessive gastrointestinal motility (1 event) in the 60-mg edoxaban OD tablet group. No serious AEs or AEs causally related with edoxaban OD tablets were observed. All AEs were confirmed to be recovered after the end of the study.

## Discussion

In this study, the subjects enrolled tended to be older (mean age, 73.0 ± 8.6 years; aged ≥75 years, 49.1%), particularly those who were allocated to the 30-mg edoxaban OD tablet groups (groups 1 and 3).

For the questionnaires regarding the current medication, 86.8% of the subjects reported no difficulty taking the current medication (question 1), 93.4% reported no difficulty identifying the drugs (question 2), and 78.3% reported taking 3 or more drugs (including edoxaban film-coated tablets) every morning (question 3), which indicates that most of the patients in this study perceived no difficulty taking the current medication including edoxaban film-coated tablets and had been taking 3 or more drugs.

In question 4 (degree of satisfaction with edoxaban OD tablets), more than half (52.8%) of the patients selected “no difference.” However, considering that “satisfactory” was selected by 34.9% in the group that included many patients who perceived no complaint about taking the current medication, the degree of satisfaction with edoxaban OD tablets was considered to be relatively high. The percentage of patients who selected “unsatisfactory” was higher in the group that ingested the drug without water than in the group with water. This result seems to reflect the fact that the rating of taste/flavor (question 7) and ease of ingestion (question 8) was frequently lower in the group that ingested the drug without water. Furthermore, the responses suggest that the patients who usually ingested drugs with water had not been accustomed to taking drugs without water.

In question 5 (ease in identifying edoxaban OD tablets), “quite difficult to identify” was selected most frequently (30.5%), but “quite easy to identify” or “easier to identify than the current edoxaban film-coated tablet” was selected by 26.7 and 27.6% of the respondents, respectively. Thus, the answers to this question were equally divided among the opposing responses. This response probably reflected the fact that the figures printed on the tablets were considered too small for some patients to read.

In question 6 (size of edoxaban OD tablets), the percentage of those who selected “slightly large” (59.2%) or “slightly small” (37.8%) was 97%, which suggests that the size of these tablets was acceptable on the whole.

In question 7 (taste and flavor at the time of edoxaban OD tablet disintegration), the percentage of selecting “very good” or “relatively good” was 81.5%. Of the 17 patients who selected “relatively bad,” 15 answered “tolerable,” which suggests no problem with the taste/flavor of this product. The percentage of selecting “relatively bad” was higher in the group that ingested edoxaban OD tablets without water than in the group that ingested it with water. The rating tended to be higher in the group that ingested edoxaban OD tablets with water. The patients who selected “good” for the taste/flavor tended to select “easier to take” in question 8, which suggests that the taste or flavor affected the perceived ease of OD tablet ingestion.

In question 8 (ease of taking edoxaban OD tablets), “no difference” was selected most frequently (42.9%), but the percentage with a favorable impression (“easier to take”) was higher than the percentage with a bad impression, which suggests that edoxaban OD tablets were easier to take. Since the percentage of selecting “difficult to take” was higher in the group that ingested edoxaban OD tablets without water than in the group that ingested edoxaban OD tablets with water, the rating tended to be higher in the group that ingested edoxaban OD tablets with water.

In question 9 (convenience of taking edoxaban OD tablets), “no difference” was selected most frequently (45.3%). However, the percentage of patients selecting “more convenient” was also high (52.8%), which suggests that edoxaban OD tablets were perceived as more convenient than the existing edoxaban film-coated tablets.

In question 10 (ease of continuation to take edoxaban OD tablets), “no difference” was selected most frequently (56.6%). However, the percentage of patients who selected “easier to continue” was relatively high (38.7%), which suggests that edoxaban OD tablets tented to be perceived as easy to continue.

In question 11 (reliability of edoxaban OD tablets), “no difference” was selected most frequently (51.9%). As the only other answer was “more reliable” (indicating a favorable impression), edoxaban OD tablets were considered reliable for reasons such as printing the product name on the tablets.

In question 12 (desire a change to edoxaban OD tablets), 49.5% of the respondents selected “desire to change” and approximately the same percentage selected “no desire to change.” Thus, 2 opposing answers were selected by a similar percentage. The desire to change tended to be more common in the group that ingested edoxaban OD tablets with water than in the group that ingested the edoxaban OD tablet without water. The reason for selecting “desire to change” was “easier to take” in most patients, probably reflecting sufficient understanding of the features of OD tablets. The reasons for selecting “no desire to change” were as follows: “water is needed when taking it with other drugs,” “I have other drugs to take simultaneously,” “it is easier to take with water,” and others. These answers suggest that the features of OD tablets (can be taken with water simultaneously with other drugs) had not been sufficiently understood by these patients.

In this study, 90% of the patients had been routinely taking 2 or more tablets, and most of the patients seemed to have been taking edoxaban tablets with water simultaneously with other drugs. For this reason, taking edoxaban OD tablets with water seems to be closer to the actual clinical setting for these patients. This study shows that the degree of satisfaction, taste/flavor, and ease of ingestion were rated higher in the group that ingested the drug with water, which suggests that the advantages of edoxaban OD tablets are better manifested when it is taken with water. As a result, it is inferred that many of the patients desired a change to edoxaban OD tablets in the group taking edoxaban OD tablets with water.

Generally, OD tablets are more advantageous for elderly patients and patients with impaired swallowing function due to cerebral infarction or other reasons. Therefore, we conducted a subgroup analysis according to the ages (< 75 and ≥ 75 years) and presence/absence of difficulty of taking current medications. In patients aged ≥75 years, the rating of reliability and percentage of desiring a change were low, with no item recording a high rating. In patients who perceived difficulty of taking current medications, the percentage of desiring a change to edoxaban OD tablets was as high as 76.9%. These results suggest that the advantages of edoxaban OD tablets were rated high by the patients who perceived difficulty taking current medications.

There are some limitations in this study. The first is that the condition for drug ingestion (designed to take edoxaban OD tablet as a single agent) differed from the actual clinical setting. Most patients were taking 2 or more tablets routinely and probably took edoxaban tablets and other medications simultaneously with water. Irikura et al. [7] investigated whether there are advantages of OD tablets for patients taking multiple drugs simultaneously and reported that 40% of medical professionals (physicians, nurses, and pharmacists) perceived no advantage of OD tablets, while 30% perceived some advantages. They additionally reported that the perceived advantages included easier ingestion if at least one drug is an OD tablet or the ability to take OD tablets without water even if it is at least one of the drugs, among others. We therefore consider it necessary to evaluate the advantages of edoxaban OD tablets under conditions of multiple drug ingestion simultaneously with water identical to that in a practical clinical setting. The second is that only 2 hospitals participated in this study, and the number of patients enrolled was limited (including only 1 woman in the 60-mg edoxaban OD tablet group). Therefore, general extrapolation of the results of this study might be difficult. A further large-scale study would be needed, especially enrolling more women in the 60-mg edoxaban OD tablet group. The third is that we cannot rule out the possibility that the features of edoxaban OD tablets (can be taken with water simultaneously with other drugs) had not been understood sufficiently by the participants. Different results may be obtained if a similar study is conducted in actual clinical practice, in which patients would be adequately informed about the possibility of taking these tablets with water.

## Conclusions

A questionnaire survey was conducted to assess edoxaban OD tablets in a study population that included many patients who perceived no difficulty taking the existing film-coated tablets. The degree of satisfaction and rating of taste/flavor, ease of ingestion, convenience, ease of continuation, and reliability were high on the whole, with about half of all the patients reporting a desire to change to edoxaban OD tablets. The rating of these features was higher in the group that ingested the drug with water than in the group without water. The percentage of patients who desired a change to edoxaban OD tablet was higher among those who perceived difficulty taking current medications. Similar results were reported in past studies of sensory evaluation for other OD tablets [4–6]. Moreover, a change to OD tablets from existing standard tablets has been reported to increase medication adherence to dosing instructions, resulting in better treatment responses [2, 3]. We may therefore expect that the use of edoxaban OD tablets will improve the patient adherence to medication and outcomes.

## Additional file

Additional file 1: **Table S1.** Results of the sensory evaluation questionnaire about edoxaban OD tablets shown by dose level (30 mg/60 mg) and with/without water. (DOCX 24 kb)

## Abbreviations

ADR: Adverse drug reaction
AE: Adverse event
DOAC: Direct oral anticoagulant
OD: Orally disintegrating
