# Supplementary material for: Sensory evaluation of edoxaban orally disintegrating tablets: an open-label interventional study (secondary publication)
Source: Thromb J. 2019 Feb 15;17:3. doi: 10.1186/s12959-019-0192-x (PMC6376672; doi:10.1186/s12959-019-0192-x)
Supplement: Supplementary file 1 — Table S1. Results of the sensory evaluation questionnaire about edoxaban OD tablets shown by dose level (30 mg/60 mg) and with/without water. (DOCX 24 kb) [file 12959_2019_192_MOESM1_ESM.docx]

**Table S1** Results of the sensory evaluation questionnaire about edoxaban OD tablets shown by dose level (30 mg/60 mg) and with/without water

|  |  | Group 1 + 3  (30 mg)^c^ | Group 2 + 4  (60 mg)^d^ | Group 1 + 2 (without water) | Group 3 + 4 (with water) |
| --- | --- | --- | --- | --- | --- |
|  |  | n (%) | n (%) | n (%) | n (%) |
| Question 1: Difficulty in ingestion (current) | Yes | 11 (17.2) | 3 (7.1) | 8 (15.1) | 6 (11.3) |
|  | No | 53 (82.8) | 39 (92.9) | 45 (84.9) | 47 (88.7) |
| Question 2: Difficulty in identifying (current) | Yes | 5 (7.8) | 2 (4.8) | 2 (3.8) | 5 (9.4) |
|  | No | 59 (92.2) | 40 (95.2) | 51 (96.2) | 48 (90.6) |
| Question 3: Number of drugs taken simultaneously | 1 type^a^ | 6 (9.4) | 4 (9.5) | 4 (7.6) | 6 (11.3) |
|  | 2 types | 8 (12.5) | 5 (11.9) | 10 (18.9) | 3 (5.7) |
|  | ≥3 types | 50 (78.1) | 33 (78.6) | 39 (73.6) | 44 (83.0) |
| Question 4: Degree of satisfaction | Unsatisfactory | 6 (9.4) | 7 (16.7) | 12 (22.6) | 1 (1.9) |
|  | No difference | 36 (56.3) | 20 (47.6) | 21 (39.6) | 35 (66.0) |
|  | Satisfactory | 22 (34.4) | 15 (35.7) | 20 (37.7) | 17 (32.1) |
| Question 5: Ease in identifying | Quite easy | 14 (21.9) | 14 (34.2) | 13 (25.0) | 15 (28.3) |
|  | Easier^b^ | 19 (29.7) | 10 (24.4) | 15 (28.9) | 14 (26.4) |
|  | More difficult^b^ | 8 (12.5) | 8 (19.5) | 9 (17.3) | 7 (13.2) |
|  | Quite difficult | 23 (35.9) | 9 (22.0) | 15 (28.9) | 17 (32.1) |
| Question 6: Size | Very large | 1 (1.7) | 1 (2.6) | 1 (2.1) | 1 (2.0) |
|  | Slightly large | 29 (49.2) | 29 (74.4) | 26 (55.3) | 32 (62.8) |
|  | Slightly small | 28 (47.5) | 9 (23.1) | 19 (40.4) | 18 (35.3) |
|  | Very small | 1 (1.7) | 0 (0.0) | 1 (2.1) | 0 (0.0) |
| Question 7: Taste at the time of disintegration | Very good | 10 (18.2) | 6 (16.2) | 6 (12.2) | 10 (23.3) |
|  | Relatively good | 35 (63.6) | 24 (64.9) | 28 (57.1) | 31 (72.1) |
|  | Relatively bad | 10 (18.2) | 7 (18.9) | 15 (30.6) | 2 (4.7) |
|  | Very bad | 0 (0.0) | 0 (0.0) | 0 (0.0) | 0 (0.0) |
| Question 8: Ease of ingestion (comparison) | Quite easy to take | 16 (25.4) | 5 (11.9) | 10 (18.9) | 11 (21.2) |
|  | Slightly easier to take | 14 (22.2) | 8 (19.1) | 9 (17.0) | 13 (25.0) |
|  | No difference | 24 (38.1) | 21 (50.0) | 18 (34.0) | 27 (51.9) |
|  | Slightly more difficult to take | 8 (12.7) | 8 (19.1) | 15 (28.3) | 1 (1.9) |
|  | Quite difficult to take | 1 (1.6) | 0 (0.0) | 1 (1.9) | 0 (0.0) |
| Question 9: Convenience | Much more convenient | 10 (15.6) | 8 (19.1) | 9 (17.0) | 9 (17.0) |
|  | Slightly more convenient | 24 (37.5) | 14 (33.3) | 15 (28.3) | 23 (43.4) |
|  | No difference | 28 (43.8) | 20 (47.6) | 27 (50.9) | 21 (39.6) |
|  | Slightly less convenient | 2 (3.1) | 0 (0.0) | 2 (3.8) | 0 (0.0) |
|  | Much less convenient | 0 (0.0) | 0 (0.0) | 0 (0.0) | 0 (0.0) |
| Question 10: Ease in continuation | Much easier to continue | 8 (12.5) | 5 (11.9) | 7 (13.2) | 6 (11.3) |
|  | Slightly easier to continue | 19 (29.7) | 9 (21.4) | 12 (22.6) | 16 (30.2) |
|  | No difference | 34 (53.1) | 26 (61.9) | 29 (54.7) | 31 (58.5) |
|  | Slightly less easy to continue | 3 (4.7) | 2 (4.8) | 5 (9.4) | 0 (0.0) |
|  | Much less easy to continue | 0 (0.0) | 0 (0.0) | 0 (0.0) | 0 (0.0) |
| Question 11: Reliability | Much more reliable | 9 (14.1) | 12 (28.6) | 8 (15.1) | 13 (24.5) |
|  | Slightly more reliable | 20 (31.3) | 10 (23.8) | 16 (30.2) | 14 (26.4) |
|  | No difference | 35 (54.7) | 20 (47.6) | 29 (54.7) | 26 (49.1) |
|  | Slightly less reliable | 0 (0.0) | 0 (0.0) | 0 (0.0) | 0 (0.0) |
|  | Much less reliable | 0 (0.0) | 0 (0.0) | 0 (0.0) | 0 (0.0) |
| Question 12: Desire to change | Desire to change | 31 (54.4) | 16 (42.1) | 18 (38.3) | 29 (60.4) |
|  | No desire to change | 26 (45.6) | 22 (57.9) | 29 (61.7) | 19 (39.6) |

^a^Edoxaban film-coated tablet only; ^b^compared with the current medication; ^c^edoxaban OD tablet 30 mg; ^d^edoxaban OD tablet 60 mg.

*OD* orally disintegrating
